# Low levels of the protein TRAIL are associated with illness severity in children with suspected infection

**DOI:** 10.3389/fped.2026.1707389

**Published:** 2026-06-03

**Authors:** Maya Youran Kimhi, Tomer Lamhoot, Mor Bar-On, Vered Nir, Adi Klein

**Affiliations:** 1 Rappaport Faculty of Medicine, Technion Institute of Technology, Haifa, Israel; 2 MeMed, Tirat Carmel, Israel; 3 Department of Pediatrics, Hillel Yaffe Medical Center, Hadera, Israel

**Keywords:** host response, illness severity, infection, pediatrics, TRAIL protein

## Abstract

**Introduction:**

Tumor Necrosis Factor-Related Apoptosis-Inducing Ligand (TRAIL) has a potential role in the immune response to infections. This study investigated the association between serum TRAIL levels and illness severity in children with suspected infection.

**Methods:**

A retrospective *post hoc* analysis was conducted in children aged 4 months to 18 years who underwent MeMed BV testing during emergency department evaluation for suspected acute infection. Patients were categorized by serum TRAIL levels using a prespecified cutoff of ≤40 pg/mL vs. >40 pg/mL. Clinical and demographic data were compared between groups, with illness severity at presentation as the primary outcome.

**Results:**

The analysis included 104 children, with 52 children in each TRAIL group. Children with TRAIL levels ≤40 pg/mL had longer hospitalization duration (4.06 ± 3.5 days vs. 2.50 ± 3.5 days, *p* = 0.026), higher pulse rates (147.9 ± 24.2 bpm vs. 135.5 ± 25.1 bpm, *p* = 0.012), and more abnormal white blood cell counts (67.3% vs. 36.5%, *p* = 0.003). They also had higher maximal C-reactive protein levels [128.6 [55.7–180.7] mg/L vs. 22.5 [4.4–56.2] mg/L, *p* < 0.001] and were more likely to receive antibiotics (85% vs. 42%, *p* < 0.001) and to require changes in antibiotic therapy (58% vs. 13.5%, *p* < 0.001). Illness severity differed significantly between groups, with severe illness observed in 44.2% of children with TRAIL ≤40 pg/mL compared with 9.6% in those with TRAIL >40 pg/mL (*p* < 0.001).

A TRAIL cutoff of ≤40 pg/mL showed moderate discriminatory performance for severe illness (AUROC 0.73), with 82.1% sensitivity, 61.8% specificity, PPV 44.2%, and NPV 90.4%.

**Discussion:**

Lower serum TRAIL levels in children were associated with more severe illness, as evidenced by extended hospital stays, increased antibiotic use, and more severe clinical presentations. These findings suggest an association between low TRAIL levels and illness severity, although differences in infection etiology between groups should be considered.

## Introduction

Physicians in pediatric emergency departments and wards face critical challenges in detecting early deterioration in children (1, 2). In current practice, clinicians integrate bedside assessment with physiological parameters and laboratory findings, and in some settings this is supported by structured early warning tools such as the Manchester Triage System and Pediatric Early Warning Scores (PEWS) (1, 3–10).

In the current healthcare landscape, pediatric departments continue to grapple with a substantial burden and missed opportunities for early intervention (11). This not only contributes to prolonged hospitalizations without clear justification but also raises concerns about overlooking potentially critical situations that could have been identified and addressed prior to deterioration (12). As medical professionals increasingly rely on a combination of clinical examination, vital signs, and laboratory analyses to gauge illness severity and determine appropriate care, the need for improved, objective methods of early risk recognition remains evident (13–15). This need is further amplified in pediatric ED settings, where clinical deterioration is relatively uncommon, resulting in a low pretest probability. Delays in initiating appropriate therapy, including timely antibiotics when indicated, have been associated with worse outcomes in infectious syndromes (12, 13).

Tumor Necrosis Factor-Related Apoptosis-Inducing Ligand (TRAIL) is a host-response cytokine involved in immune regulation and apoptosis (16–18). Studies have shown that circulating TRAIL levels tend to rise in viral infections and decrease in bacterial infections, particularly in severe bacterial disease (15, 19, 21). TRAIL has been incorporated into host-protein approaches used to support bacterial-viral differentiation alongside clinical assessment (15, 19). Beyond etiology, lower TRAIL levels have also been reported in association with more severe systemic infection states, including severe COVID-19 (20) and sepsis/septic shock (21).

Timely host-response results, interpreted alongside clinical findings, may help reduce unnecessary antibiotic exposure (22). In addition, they may support earlier, more targeted decisions regarding disposition (discharge vs. admission) and the need for closer observation in children at risk of deterioration (15, 19). Taken together, these findings suggest that TRAIL may capture clinically relevant host-response information that could complement standard bedside assessment.

Our study aimed to enhance early identification of children at risk of developing severe illness at the initial pediatric emergency department visit by examining the association between low TRAIL levels and illness severity in children with suspected infection.

## Materials and methods

### Study population

This retrospective *post hoc* sub-analysis of the SPIRIT (NCT03075111) study was conducted at Hillel Yaffe Medical Center. We identified children aged 4 months to 18 years who presented to the pediatric emergency department between January 2015 and October 2017 and had a MeMed BV test performed as part of routine care for suspected acute infection. Suspected acute infection was defined by the treating pediatrician's clinical assessment. The low-TRAIL group included all eligible patients with serum TRAIL ≤40 pg/mL during the study period. The control group included patients with TRAIL >40 pg/mL selected from the same SPIRIT cohort and matched 1:1 to the low-TRAIL group by age and sex. Controls were identified sequentially and included once a suitable match for each case was found.

#### Inclusion criteria

Children 4 months–18 years; ED visit within the study period; MeMed BV test performed for suspected acute infection.

#### Exclusion criteria

Repeat visits of the same patient (only the first included); missing diagnosis; chronic immunodeficiency/immunosuppression if documented in the record.

### Data collection

The following parameters were collected from the hospital electronic medical record for each subject: demographic information, medical history, current symptoms, results of physical examination, and results of the MeMed BV blood test (MeMed Diagnostics Ltd, Tirat Carmel, Israel). MeMed BV is a host-response blood test that measures TRAIL, IP-10, and CRP to support differentiation between bacterial and viral infection. The test was performed at the time of emergency department evaluation as part of routine care, as documented in the electronic medical record.

### Data analysis

Descriptive statistics are reported as mean (SD) for approximately normally distributed variables, as median (IQR) for non-normally distributed variables, and as n (%) for categorical variables. Comparisons between the TRAIL groups (≤40 pg/mL vs. >40 pg/mL) were performed using the Mann Whitney U test for continuous variables and Fisher's exact test for 2 × 2 categorical variables. The association between TRAIL group and the three level illness severity classification (mild, moderate, severe) was assessed using the chi square test. All tests were two sided, and p values <0.05 were considered statistically significant. Analyses were conducted in SPSS (version 29).

Given multiple secondary comparisons, we used Bonferroni correction (*α*=0.05/k) to control the family-wise error rate.

Diagnostic accuracy analysis: We evaluated the performance of a TRAIL cutoff of ≤40 pg/mL to identify severe illness at presentation. Sensitivity, specificity, positive predictive value (PPV), and negative predictive value (NPV) were calculated. Discriminatory performance was assessed using the area under the receiver operating characteristic curve (AUROC).

### Ethics approval

The study was approved by the institutional review board and conducted in accordance with the principles of the Declaration of Helsinki. Informed consent was waived due to the retrospective nature of the study. Confidentiality of the data was maintained throughout the study.

### Research variables

We collected physiological and laboratory parameters (e.g., temperature, oxygen saturation, blood pressure, pulse, WBC, CRP) to characterize clinical presentation and management. Additionally, we captured inflammatory markers (WBC and CRP) and recorded whether antibiotics were prescribed, switched, or added to the treatment regimen. We also considered the need for intensive care, prolonged hospitalization, or mortality as indicators of severe outcomes. Etiology (bacterial vs viral) was classified as previously described in the SPIRIT study when available and was used for etiology-stratified analyses. TRAIL was analyzed both as a continuous variable and dichotomized into low and high groups using a prespecified cutoff of 40 pg/mL (≤40 vs. >40 pg/mL), selected *a priori* based on prior published TRAIL distributions in severe infectious disease states (20, 21). In adult sepsis cohorts, TRAIL levels are lower in sepsis/septic shock compared with controls, with reported values bracketing the ∼40 pg/mL range (21).

Illness severity was classified using a diagnosis-based framework (mild, moderate, severe) based on the discharge diagnosis (Table 1), derived from a validated pediatric diagnosis-based severity classification system and adapted to the diagnoses observed in our cohort (23). When multiple diagnoses were recorded, the highest severity category was assigned.

**Table 1:** Diagnosis-based illness severity classification.

Mild	Moderate	Severe
Viral infection	Pneumonia	Pyelonephritis
Gastroenteritis	Bronchiolitis	Peritonsillar abscess
colitis	RSV infection	Acute appendicitis
Otitis media	Pneumonia – Parainfluenza	Enterovirus diseases - central nervous system
Streptococcal pharyngitis	Pneumonia – Adenovirus	Acute osteomyelitis
Herpetic gingivostomatitis	Pneumonia – Mycoplasma	Meningitis
Scarlet fever	Reactive arthritis	Bacterial meningitis
Tonsillitis	Cellulitis	Acute pancreatitis
	Influenza	Pneumonia – Staphylococcus
	Fever of unknown origin	Pneumonia – Streptococcus Group B
	UTI	Influenza pneumonia
	Abscess	
	Infectious mononucleosis	

RSV, respiratory syncytial virus; UTI, urinary tract infection.

When multiple diagnoses were present, the highest severity category was assigned. Records labeled “Other/Unknown” in the clinical syndrome field were categorized according to the ICD-9 discharge diagnosis.

This study was conducted and reported in accordance with the Standards for Reporting Diagnostic Accuracy Studies (STARD) guidelines.

## Results

### Cohort characteristics

**Figure 1:**
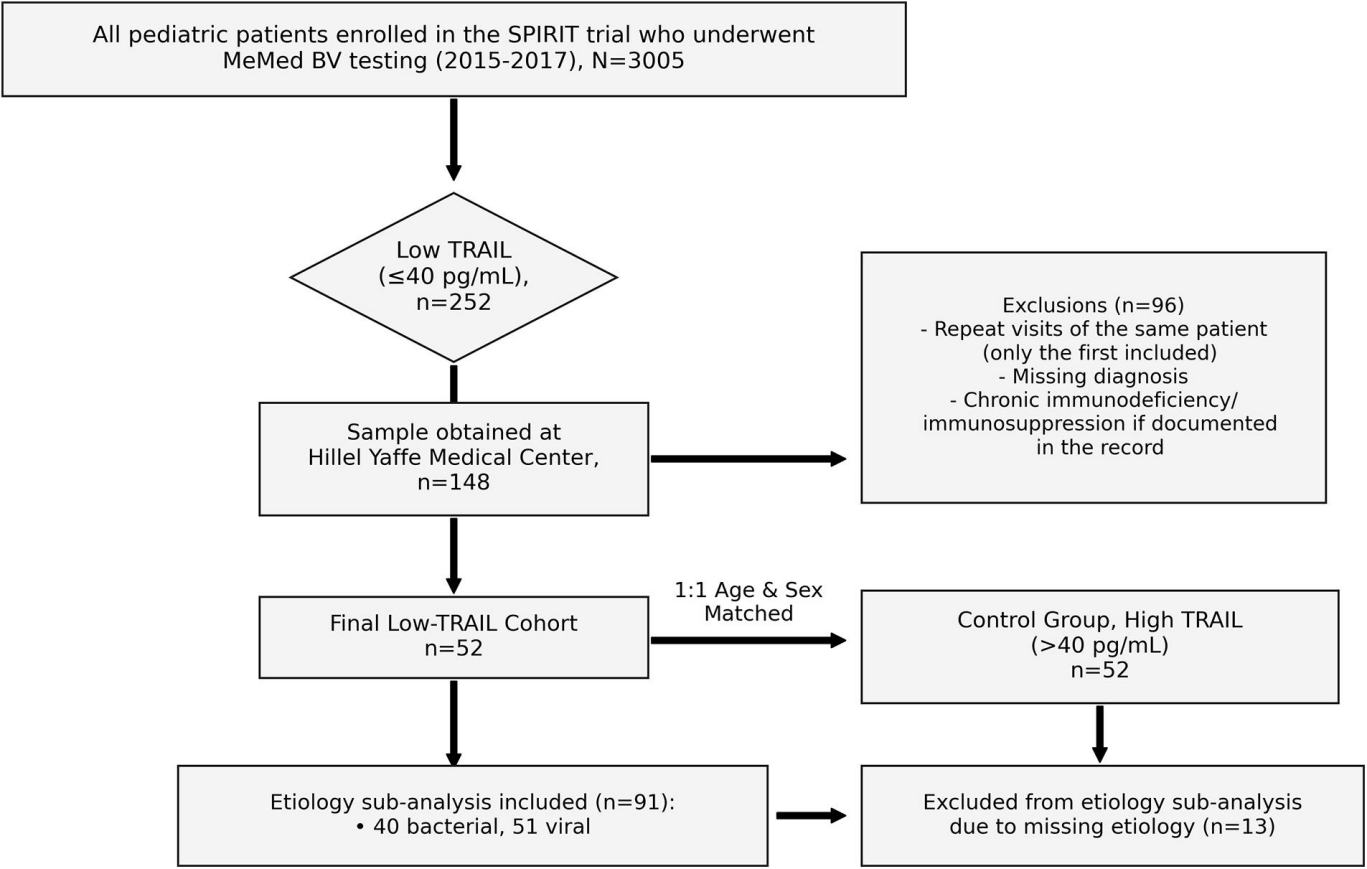
Flow chart of cohort selection and matching.

A total of 104 children presenting to the emergency department were included in this study (Figure 1). The low-TRAIL group included 52 patients with serum TRAIL ≤40 pg/mL, and the matched high-TRAIL group included 52 patients with serum TRAIL >40 pg/mL. The demographic characteristics of both groups were similar, with no significant differences observed in terms of age or gender (Table 2). The time from fever onset was not significantly different between the groups (*p* = 0.52). However, the total hospitalization duration was significantly longer in the low TRAIL levels group compared to the high TRAIL levels group (4.06 ± 3.5 days vs. 2.50 ± 3.5 days, *p* = 0.026). Pulse rate was significantly higher in the low TRAIL group compared to the high TRAIL group (147.9 ± 24.2 vs. 135.5 ± 25.1 beats per minute, *p* = 0.012).

**Table 2:** Demographic, clinical, laboratory, and management characteristics by TRAIL group.

Patient characteristic	Below 40; *n* = 52	Above 40; *n* = 52	p
Age	4.3 ± 4.01	4.3 ± 4.01	*P* = 0.99
Gender			*P* = 1.00
Female	28 (54%)	28 (54%)	
Male	24 (46%)	24 (46%)	
Time from fever onset (d)	2.5 ± 1.73	2.8 ± 3.18	*P* = 0.52
Total hospitalization duration (days)	4 ± 3.5 (4)	2.5 ± 3.5 (2)	***P*** **=** **0.026**
Maximal temperature	39.4 ± 0.95	39.1 ± 1.07	*P* = 0.088
Pulse	147.9 ± 24.2	135.5 ± 25.1	***P*** **=** **0.012**
BP systolic	100.4 ± 11.7	100.6 ± 11.0	*P* = 0.92
BP diastolic	60.9 ± 9.6	59.0 ± 8.8	*P* = 0.32
Oxygen saturation			
on room air	97.0 ± 3.2	97.6 ± 2.9	*P* = 0.34
WBC (10^9/L)			***P*** **=** **0.003**
Below 15	17 (32.7%)	33 (63.5%)	
Above 15	35 (67.3%)	19 (36.5%)	
PLT (10^9/L)			*P* = 0.72
Above 500	3 (6%)	5 (10%)	
Below 500	49 (94%)	47 (90%)	
Hb (g%)			*P* = 0.72
Above 10	47 (90%)	49 (94%)	
Below 10	5 (10%)	3 (6%)	
CRP Max (mg/L); median [IQR]	128.6 [55.7–180.7]	22.5 [4.4–56.2]	***P*** **<** **0.001**
Albumin (g/dL)	4.04 ± 0.55; *N* = 21	4.29 ± 0.28; *N* = 19	*P* = 0.076
Abx prescribed	44 (85%)	22 (42%)	***P*** **<** **0.001**
Added	11 (21%)	2 (4%)	***P*** **=** **0.015**
Changed	30 (58%)	7 (13.5%)	***P*** **<** **0.001**
Illness severity			***P*** **<** **0.001**
Severe	23 (44.2%)	5 (9.6%)	
Moderate	17 (32.7%)	16 (30.8%)	
Mild	12 (23.1%)	31 (59.6%)	

Abx, antibiotics; BP, blood pressure; CRP, C-reactive protein; d, days; Hb, hemoglobin; IQR, interquartile range; PLT, platelets; WBC, white blood cells.

### Clinical and laboratory findings

**Figure 2:**
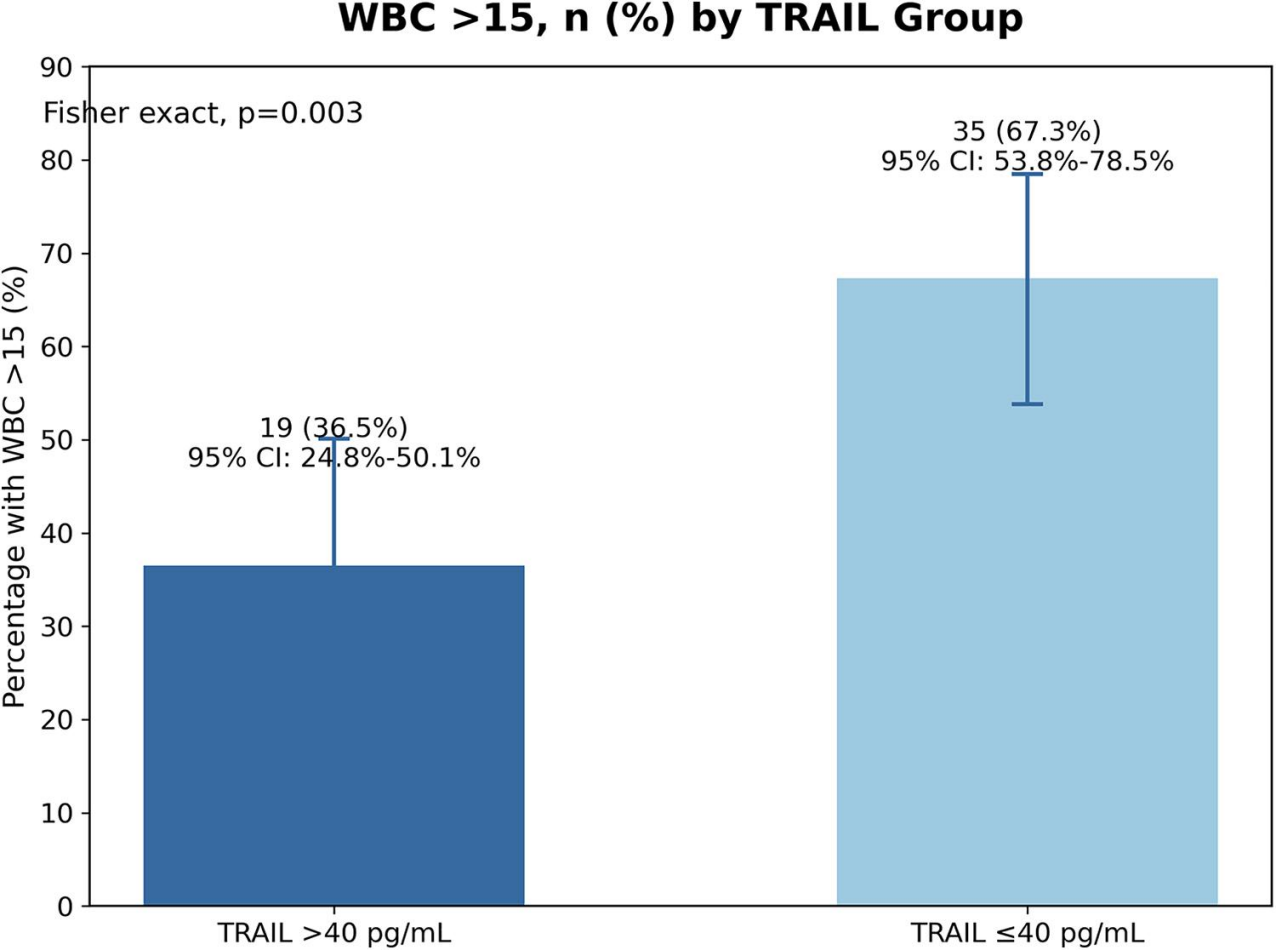
Comparison of white blood cell counts between TRAIL groups WBC, white blood cells.

In contrast, no significant differences were found in blood pressure, oxygen saturation, or maximal temperature between the groups. Abnormal white blood cell count (WBC, above 15 × 10^9/L) was significantly higher in the low TRAIL group compared to the other group (67.3% vs. 36.5%, *p* = 0.003) (Table 2; Figure 2).

**Figure 3:**
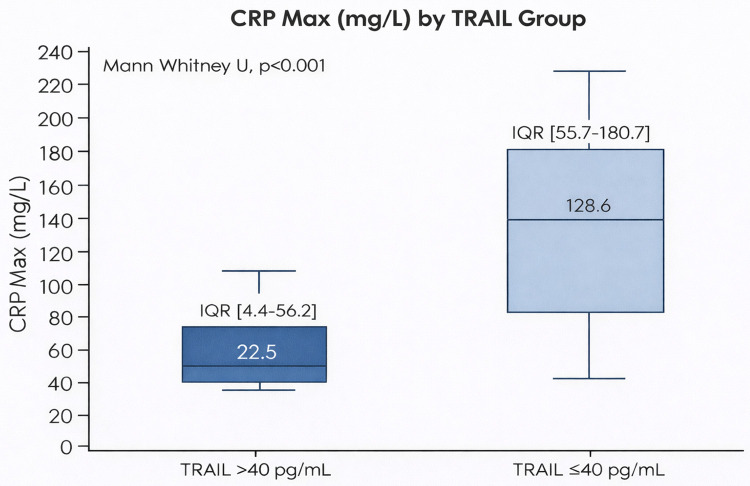
Median CRP maximal level (mg/L) comparing the two groups. CRP, C reactive protein; IQR, interquartile range.

Maximal CRP levels were significantly higher in the low TRAIL levels group compared to the high TRAIL levels group [128.6 [55.7–180.7] mg/L vs. 22.5 [4.4–56.2] mg/L, *p* < 0.001] (Table 2; Figure 3). Albumin levels were not significantly different between the groups (*p* = 0.076).

### Antibiotic treatment and illness severity

Antibiotics (Abx) were prescribed more frequently in the low TRAIL levels group compared to the other group (85% vs. 42%, *p* < 0.001). Additionally, the need for an additional antibiotic as well as the need to swap to another antibiotic was significantly more common in the low TRAIL levels group compared to the high TRAIL levels group (21% vs. 4%, *p* = 0.015% and 58% vs. 13.5%, *p* < 0.001 respectively).

**Figure 4:**
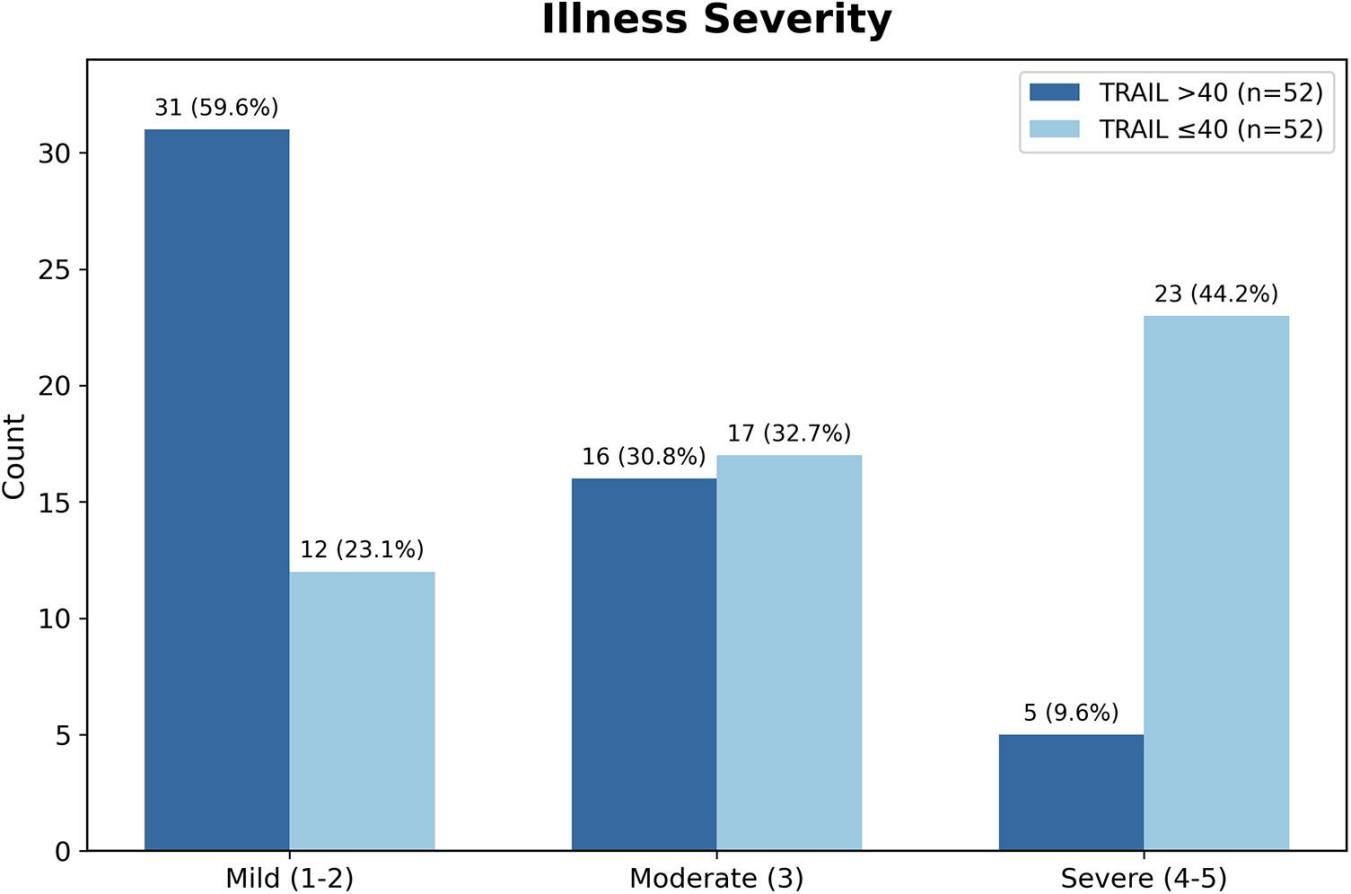
Illness severity comparison between the two groups.

The severity of illness was significantly higher in the low-TRAIL group compared to the high-TRAIL group (44.2% [23/52] vs. 9.6% [5/52] severe, 32.7% [17/52] vs. 30.8% [16/52] moderate, and 23.1% [12/52] vs. 59.6% [31/52] mild, chi-square *p* < 0.001) (Table 3; Figure 4). When comparing severe vs. mild illness (excluding the moderate category), low TRAIL was associated with markedly higher odds of severe disease (OR 11.88, 95% CI 3.67–38.46; Fisher's exact *p* < 0.001) (Table 3).

**Table 3:** Illness severity classification and primary effect size by TRAIL group.

Severity tier	TRAIL ≤40 pg/mL (*n* = 52)	TRAIL >40 pg/mL (*n* = 52)	p-value
**Mild (score 1–2)**	12 (23.1%)	31 (59.6%)	
**Moderate (score 3)**	17 (32.7%)	16 (30.8%)	
**Severe (score 4–5)**	23 (44.2%)	5 (9.6%)	<0.001^a^
Primary effect size			
**OR severe vs mild (excluding moderate)**	**11.88 (95% CI 3.67–38.46)**	reference	<0.001*

* Fisher's exact p-value for 2 × 2 (severe vs mild, excluding moderate).

a Chi-square p-value for 2 × 3 severity tiers.

### Diagnostic performance and etiology

Using the prespecified cutoff of TRAIL ≤40 pg/mL to identify severe illness (severity score 4–5), sensitivity was 82.1% and specificity was 61.8%. The positive predictive value was 44.2% and the negative predictive value was 90.4%. In receiver operating characteristic analysis using TRAIL as a continuous variable, the area under the curve was 0.73 (Table 4).

**Table 4:** Diagnostic performance of TRAIL ≤40 pg/mL for identifying severe illness at presentation.

TRAIL group	Severe (*n* = 28)	Not severe (*n* = 76)
**TRAIL ≤40 pg/mL**	23 (44.2%)	29 (55.8%)
**TRAIL >40 pg/mL**	5 (9.6%)	47 (90.4%)

Sensitivity: 82.1% Specificity: 61.8% Positive predictive value (PPV): 44.2% Negative predictive value (NPV): 90.4% Area under the receiver operating characteristic curve (AUROC): 0.73.

**Figure 5:**
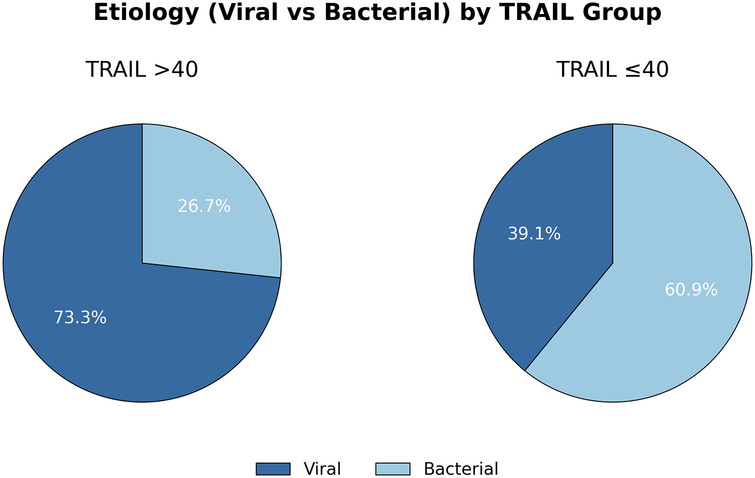
Etiology (viral vs bacterial) by TRAIL group.

Among patients with available etiologic classification (*n* = 91), bacterial infections were more common in the low-TRAIL group compared to the high-TRAIL group [60.9% [28/46] vs. 26.7% [12/45]], whereas viral infections were more common in the high-TRAIL group [73.3% [33/45] vs. 39.1% [18/46], *p* = 0.001] (Table 5, Figure 5).

**Table 5:** Etiologic classification among patients with available etiology, by TRAIL group.

Etiology (available cases)	TRAIL ≤40 pg/mL (*n* = 46)	TRAIL >40 pg/mL (*n* = 45)	p-value
**Bacterial**	28 (60.9%)	12 (26.7%)	0.0015*
**Viral**	18 (39.1%)	33 (73.3%)	
**Total with classification**	46	45	

* Fisher's exact (2 × 2).

Etiology was available for 91/104 patients.

## Discussion

In this retrospective cohort of 104 children presenting to the emergency department with suspected infection, low serum TRAIL levels (≤40 pg/mL) were strongly associated with greater illness severity. Using the predefined three-tier severity classification, children in the low-TRAIL group had substantially higher odds of severe vs. mild disease (OR 11.88, 95% CI 3.67–38.46) (Table 3). This association was supported by a consistent clinical and laboratory pattern, including higher rates of leukocytosis, markedly higher maximal CRP levels, and longer hospitalization duration.

Illness severity was defined *a priori* using a diagnosis-based severity score mapped from ICD-9 codes and grouped into three tiers for analysis: mild (scores 1–2), moderate (score 3), and severe (scores 4–5) (23). This approach allowed us to examine the relationship between TRAIL and clinically meaningful severity at presentation, rather than relying solely on isolated laboratory abnormalities.

Beyond group-level associations, we observed a clinically relevant subgroup of seven children in the low-TRAIL group who met criteria for severe illness despite not presenting with leukocytosis. Two of these children also had CRP levels below 50 mg/L. This observation suggests that TRAIL may provide additional early information during the emergency department evaluation, highlighting children who warrant closer attention even when commonly used inflammatory markers appear relatively reassuring.

TRAIL also showed a clear relationship with infection etiology in our cohort: bacterial infections were more frequent in the low-TRAIL group, whereas viral infections were more frequent in the high-TRAIL group (Table 5, Figure 5). This difference is clinically relevant, as bacterial infections are generally associated with greater illness severity and may partly contribute to the observed association. These findings are consistent with previous host-response studies in which TRAIL contributed to differentiating bacterial from viral infection as part of multi-protein diagnostic approaches (15, 19). They are also in line with reports linking lower circulating TRAIL levels with more severe infectious disease states, including severe COVID-19 and sepsis/septic shock (20, 21). Because bacterial infections were more frequent in the low-TRAIL group, infection etiology should be considered when interpreting the association between low TRAIL and illness severity. Larger cohorts will be needed to evaluate the relationship between TRAIL and severity within bacterial and viral subgroups separately.

Nevertheless, the consistent associations between low TRAIL levels and multiple clinical and laboratory severity indicators, including higher CRP levels, leukocytosis, and longer hospitalization duration, support a potential role of TRAIL as a marker of clinically relevant host response beyond etiology alone. We used a prespecified cutoff of 40 pg/mL, consistent with previously reported TRAIL levels in severe infectious disease states (20, 21). At the same time, low TRAIL aligned with greater clinical severity in our cohort, suggesting potential value for emergency department risk stratification beyond etiologic classification alone.

In secondary performance analyses, the prespecified threshold of TRAIL ≤40 pg/mL showed a sensitivity of 82.1% for severe illness at presentation (severity score 4–5), with a negative predictive value of 90.4% (Table 4). These findings suggest that higher TRAIL levels may be reassuring with respect to severe disease at the initial evaluation. In addition, the AUROC of 0.73 supports a moderate ability of TRAIL to discriminate severe from non-severe illness; larger prospective cohorts will be important to refine these estimates and evaluate how TRAIL performs alongside standard clinical assessment.

Several limitations should be considered. First, the retrospective design limits causal inference and may introduce selection bias, including dependence on clinician-driven testing and admission decisions. Second, the cohort included a heterogeneous mix of infectious diagnoses, which may enhance generalizability but limits conclusions for specific syndromes. Third, etiologic classification was available for a subset of patients, and some laboratory measures were not available for all participants; analyses were therefore based on available data. In addition, the relatively small sample size within etiologic subgroups limited our ability to perform stratified analyses (viral vs bacterial), and such analyses may be underpowered. Finally, this was a single-center study, and external validation in independent pediatric cohorts is needed. Prospective studies should evaluate whether integrating TRAIL into early risk stratification can improve disposition decisions and outcomes without increasing unnecessary interventions.

In conclusion, low TRAIL levels at emergency department presentation were strongly associated with severe illness in children with suspected infection. Low TRAIL marked a higher-risk phenotype based on our severity classification and hospitalization outcomes, and it identified a subset of severely ill children even when routine inflammatory markers were not markedly abnormal. These findings support further prospective evaluation of TRAIL as an adjunctive tool for early pediatric risk stratification in the emergency department.

## Data Availability

The datasets analyzed for this study are not publicly available due to privacy and ethical restrictions. Requests to access the datasets should be directed to Maya Youran Kimhi, mkimchi18@gmail.com.
